# Role of Epithelial-Mesenchyme Transition in *Chlamydia* Pathogenesis

**DOI:** 10.1371/journal.pone.0145198

**Published:** 2015-12-17

**Authors:** Joseph U. Igietseme, Yusuf Omosun, Olga Stuchlik, Matthew S. Reed, James Partin, Qing He, Kahaliah Joseph, Debra Ellerson, Brigid Bollweg, Zenas George, Francis O. Eko, Claudiu Bandea, Hsi Liu, Genyan Yang, Wun-Ju Shieh, Jan Pohl, Kevin Karem, Carolyn M. Black

**Affiliations:** 1 National Center for Emerging Zoonotic and Infectious Diseases, Centers for Disease Control & Prevention (CDC) Atlanta, GA 30333 United States of America; 2 Department of Microbiology, Biochemistry & Immunology, Morehouse School of Medicine, Atlanta, GA 30310 United States of America; The University of Texas at San Antonio, UNITED STATES

## Abstract

*Chlamydia trachomatis* genital infection in women causes serious adverse reproductive complications, and is a strong co-factor for human papilloma virus (HPV)-associated cervical epithelial carcinoma. We tested the hypothesis that Chlamydia induces epithelial-mesenchyme transition (EMT) involving T cell-derived TNF-alpha signaling, caspase activation, cleavage inactivation of dicer and dysregulation of micro-RNA (miRNA) in the reproductive epithelium*;* the pathologic process of EMT causes fibrosis and fertility-related epithelial dysfunction, and also provides the co-factor function for HPV-related cervical epithelial carcinoma. Using a combination of microarrays, immunohistochemistry and proteomics, we showed that chlamydia altered the expression of crucial miRNAs that control EMT, fibrosis and tumorigenesis; specifically, miR-15a, miR-29b, miR-382 and MiR-429 that maintain epithelial integrity were down-regulated, while miR-9, mi-R-19a, miR-22 and miR-205 that promote EMT, fibrosis and tumorigenesis were up-regulated. Chlamydia induced EMT *in vitro* and *in vivo*, marked by the suppression of normal epithelial cell markers especially E-cadherin but up-regulation of mesenchymal markers of pathological EMT, including T-cadherin, MMP9, and fibronectin. Also, *Chlamydia* upregulated pro-EMT regulators, including the zinc finger E-box binding homeobox protein, ZEB1, Snail1/2, and thrombospondin1 (Thbs1), but down-regulated anti-EMT and fertility promoting proteins (i.e., the major gap junction protein connexin 43 (Cx43), Mets1, Add1Scarb1 and MARCKSL1). T cell-derived TNF-alpha signaling was required for chlamydial-induced infertility and caspase inhibitors prevented both infertility and EMT. Thus, chlamydial-induced T cell-derived TNF-alpha activated caspases that inactivated dicer, causing alteration in the expression of reproductive epithelial miRNAs and induction of EMT. EMT causes epithelial malfunction, fibrosis, infertility, and the enhancement of tumorigenesis of HPV oncogene-transformed epithelial cells. These findings provide a novel understanding of the molecular pathogenesis of chlamydia-associated diseases, which may guide a rational prevention strategy.

## Introduction

*Chlamydia trachomatis* genital infection is the most common bacterial STD worldwide. The complications include pelvic inflammatory disease (PID), ectopic pregnancy and tubal factor infertility (TFI). Also, chlamydia is a risk factor for human papilloma virus (HPV)-associated cervical epithelial dysplasia (intraepithelial neoplasia) and cervical carcinoma [1]. Apart from the clinical evidence of tubal obstruction attributed to inflammation-driven fibrosis [2], the molecular pathogenesis of genital chlamydial complications or its co-factor role in HPV-related cervical carcinoma remains unclear. However, recent reports revealed that chlamydial genital infection caused significant alterations in host regulatory micro-RNA (miRNA) expression profiles in the reproductive system [3–5]. MiRNAs are an evolutionarily conserved, short (~22 nucleotides) non-coding RNAs that posttranscriptionally regulate gene expression by binding to complementary 3’UTR of mRNAs, resulting in mRNA degradation, translational repression or occasionally enhancement. Physiologically, miRNAs regulate gene expression during cellular differentiation, reproduction, development, maintenance of cellular integrity, functions and normal metabolism, as well as in pathologic fibrosis and oncogenesis, accounting for approximately 30% of mammalian gene expression [6]. Furthermore, *Chlamydia*-induced disruption of reproductive tissue miRNAs expression was associated with the activation of specific caspases that target Dicer [3], a ribonuclease III critical in the biogenesis of miRNAs and siRNAs [6]. The miRNAs altered during chlamydial infection included those that regulate epithelial functional integrity [7]. This suggested that chlamydia may induce epithelial-mesenchyme transition (EMT) [7–9] that could affect fertility-related reproductive epithelial functions and may also promote epithelial cell transformation and/or tumor progression.

EMT is an important miRNA-regulated biological process that converts normal polarized, cobble-stone-like epithelial cells into fibroblastic (elongated) mesenchyme cells and consequently alters epithelial integrity and functions. Mesenchymal cells exhibit enhanced motility and migratory capacity, invasive capability, higher resistance to senescence and apoptosis, and increased production of extracellular matrix (ECM) components [7,8]. EMT represents an important phase in development, differentiation, inflammation, fibrogenesis and tumorigenesis [7,10]. Specifically, EMT is involved in cellular differentiation for specialized tissue formation and organogenesis during embryogenesis and development; production of myofibroblasts from epithelial cells during fibrosis, tissue repair or wound healing after injury; secretion of pro-inflammatory molecules by epithelial cells during inflammation; and initiating the invasive and metastatic behavior of epithelial cancers [7]. Thus, EMT plays a major role in organ and tissue fibrotic diseases, such as pulmonary, renal, and hepatic fibrosis [9]; however, its role in the tubal fibrosis associated with genital chlamydial disease [2] has not been investigated. EMT converts normal epithelial parenchymal tissue architecture into non-functional scar tissue during fibrosis [11] and alter the normal differentiated functions of epithelial cells (i.e., secretory, barrier and transport) [12–14]. Mechanistically, EMT inducers, including growth factors, hormones (e.g., estrogen) and proinflammatory cytokines (TNF-alpha and TGF-beta) and their receptors, activate intracellular signaling pathways (e.g., the Smad, PI3K/ERK and the Wnt/beta-catenin pathways) that disrupt the fine miRNA regulatory processes maintaining the balance of E-cadherin on epithelial cells through interactions between miRNAs and key transcription factors (TFs) [7–9]. Three families of TFs {SNAIL1/2 (i.e., SNAIL1 and SLUG), TWIST, and zinc-finger E-box-binding, ZEB1} are the principal mediators of EMT; these TFs target the repression of epithelial cadherin and other normal epithelial cell markers such as beta-catenin and connexin proteins that are components of adherens, tight and gap junctions, causing loss of adherens junctions and cell-cell adhesion. Loss of E-cadherin is the hallmark of EMT. Concomitantly, the EMT mediators activate key mesenchymal genes encoding mesenchymal cell markers, including N- and/or T-cadherin (cell-cell adhesion), vimentin (an intermediate filament protein that regulates cell motility), fibronectin 1 (cell growth and migration) and matrix metalloproteinases. Changes in cellular gene expression pattern of the RHO GTPases drive molecular changes leading to reorganization of cytoskeletal architecture, loss of epithelial tight junctions and apical-basolateral polarity, acquisition of a front-rear polarity, changes in cell shape with increased protrusions, and greater cellular motility for an invasive capability [7,9]. The key miRNA signature profile of EMT is the down-regulation of members of the miR-200 family, especially miR-429, which normally exerts suppressive function on negative regulators of E-cadherin (e.g., ZEB1, TWIST and SNAIL1/2). In addition, there is the down-regulation of tumor suppressors, such as p53, and upregulation of a number of tumor promoters that involved in tumorigenesis and metastasis. The altered expression and dysregulation of key miRNAs underlying these changes are caused by the action of EMT mediators such as ZEB1 [15].

EMT-inducing proinflammatory cytokines are also key mediators of endometritis, salpingitis, tubal fibrosis and other inflammatory sequelae of genital chlamydial infection [16], although the role of miRNA-driven EMT in the pathologic processes that culminates in reproductive fertility-related complications was unknown. Besides, the contribution of infection-induced EMT to fibrosis or invasive and metastatic carcinoma has not been studied. However, chlamydia induces fibrosis [2] and is a strong co-factor for HPV-related cervical carcinoma [1] although the molecular basis has been an enigma. Since chlamydial genital infection could cause significant alterations in reproductive system miRNA expression profiles [3–5], we tested the hypothesis that the molecular basis of chlamydia-induced infertility and chlamydia as a co-factor in HPV-induced cervical epithelial carcinoma is the induction of EMT through miRNA dysregulation. The result indicated that EMT is a caspase-driven pathological process induced during chlamydial infection. EMT induction by chlamydia and the consequent alteration of epithelial functional integrity may provide a molecular basis for reproductive infertility or the co-factor role in HPV-induced cervical epithelial neoplasia and invasive carcinoma.

## Results

### Chlamydial genital infection caused a sustained alteration of key miRNAs that control the functional integrity of epithelial cells

We recently showed that chlamydial genital infection leading to infertility or other pathologies was associated with the cleavage inactivation of the key miRNA-synthesis enzyme Dicer [3]. Also, we and others have demonstrated the altered expression of several miRNAs in the reproductive tissues of chlamydia-infected animals [3–5]. We investigated how the altered miRNAs drove the pathogenesis of *Chlamydia*-induced reproductive diseases or provide a mechanism for the co-factor role of *Chlamydia* in HPV-related reproductive epithelial carcinoma. It was hypothesized that chlamydial genital infection will induce the altered expression of miRNAs that control the functional integrity and homeostasis of the reproductive epithelium. We performed a detailed quantitative comparative analysis of miRNAs from the oviducts of infected (infertile) and non-infected (fertile) animals; we followed the miRNA dysregulation over a period of time during which the pathophysiological processes associated with chlamydial infection do manifest; and we employed *in silico* functional analysis to determine if there were any established relationships between the dysregulated miRNAs and the known complications of chlamydia infection, including fibrosis, loss of epithelial functional integrity relating to reproduction, and promotion of epithelial neoplasia. Results presented in Table 1 are a list of *Chlamydia*-induced differentially expressed miRNAs with at least 2-fold down- or up-regulation from oviducts harvested between 1 and 12 wk (80 days) post-infection, relative to non-infected fertile animals. Both gene expression pathway analysis, involving loss-of-function assays, and *in silico* database search for miRNA targets in the relevant molecular pathways they regulate (http://www.microrna.org/microrna/home.do; http://targetscan.org/), have established the functional significance of several of these miRNAs.

**Table 1:** *Chlamydia*-Induced Sustained Differentially Expressed miRNAs (http://www.microrna.org/microrna/home.do; http://targetscan.org/).

#	Differentially-4Expressed miRNA	Fold-Change: Down (-)-/Up (+) Regulation	Biological Function Relating to Epithelial Functional Integrity and Tumorigenesis	Refs
1	miR-10b	~ 2-fold (-)	Tumor control	[105]
**2**	**miR-15a**	**~ 5-fold (-)**	**EMT control**	**[17,18]**
**3**	**miR-16**	**~ 3-fold (-)**	**EMT control**	**[17,18]**
4	miR-17-3p	~ 2-fold (-)	EMT/tumor control	[83]
5	miR-21	~ 4-fold (-)	Cell growth	
6	miR-24	~ 3-fold (-)		
7	miR-25	~ 2-fold (-)		
8	miR-27a	~ 4-fold (-)		
**9**	**miR-29a**	**~ 6-fold (-)**	**Fibrosis and tumor suppressor**	**[19,22]**
**10**	**miR-29b**	**~ 6-fold (-)**	**Fibrosis and tumor suppressor**	**[19,22]**
11	miR-30a-3p	~ 2-fold (-)	Tumor suppressor, apoptosis, replication	
12	miR-30a-5p	~ 2-fold (-)	Tumor suppressor, apoptosis, replication	
13	miR-32	~ 2-fold (-)		
14	miR-100	~ 2-fold (-)		
15	miR-101-1	~ 2-fold (-)		
16	miR-103	~ 2-fold (-)		
17	miR-131	~ 2-fold (-)		
18	miR-136	~ 2-fold (-)		
19	miR-142-3p	~ 2-fold (-)		
20	miR-143	~ 3-fold (-)		
21	miR-185	~ 2-fold (-)		
22	miR-190	~ 2-fold (-)		
23	miR-191	~ 2-fold (-)		
24	miR-198	~ 3-fold (-)		
25	miR-200a	~ 2-fold (-)	EMT/tumor control	[15,24,25]
26	miR-200b	~ 2-fold (-)	EMT/tumor control	
27	miR-200c	~ 2-fold (-)	EMT/tumor control	
28	miR-203	~ 2-fold (-)	EMT/tumor control	[10]
29	miR-204	~ 2-fold (-)	EMT/tumor control	[10]
30	miR-210	~ 2-fold (-)		
31	miR-214	~ 2-fold (-)		
32	miR-221	~ 3-fold (-)		
33	miR-296	~ 2-fold (-)		
34	miR-340	~ 2-fold (-)		
35	miR-372	~ 2-fold (-)		
36	miR-376a#	~ 2-fold (-)		
37	miR-376b	~ 3-fold (-)		
38	miR-379	~ 2-fold (-)		
**39**	**miR-382**	**~ 5-fold (-)**	**Epithelial integrity**	**[15]**
40	miR-384	~ 2-fold (-)		
**41**	**miR-409-5p**	**~ 3-fold (-)**	**Epithelial integrity**	**[15]**
42	miR-411	~ 2-fold (-)		
43	miR-412	~ 4-fold (-)		
44	miR-422a	~ 5-fold (-)		
**45**	**miR-429**	**~ 8-fold (-)**	**EMT/tumor control**	**[15,24,25]**
46	miR-432	~ 3-fold (-)		
**47**	**miR-9**	**~ 6-fold (+)**	**EMT inducer. Oncogenic**	**[8,26,27]**
**48**	**miR-19a**	**~ 2-fold (+)**	**EMT/oncogenic**	
**49**	**miR-22**	**~ 3-fold (+)**	**Oncogenic. EMT inducer**	**[28,29]**
50	miR-122a	~ 2-fold (+)		
51	miR-124a	~ 3-fold (+)		
52	miR-199b	~ 3-fold (+)		
53	miR-224	~ 2-fold (+)		
54	miR-324-5p	~ 3-fold (+)		
55	miR-331	~ 4-fold (+)		
56	miR-342	~ 2-fold (+)		
57	miR-449	~ 4-fold (+)		
58	miR-449b	~ 4-fold (+)		
59	miR-451	~ 2-fold (+)	Promotes cell migration & tumorigenesis	[81]
60	miR-452	~ 4-fold (+)		

**Fig 1:**
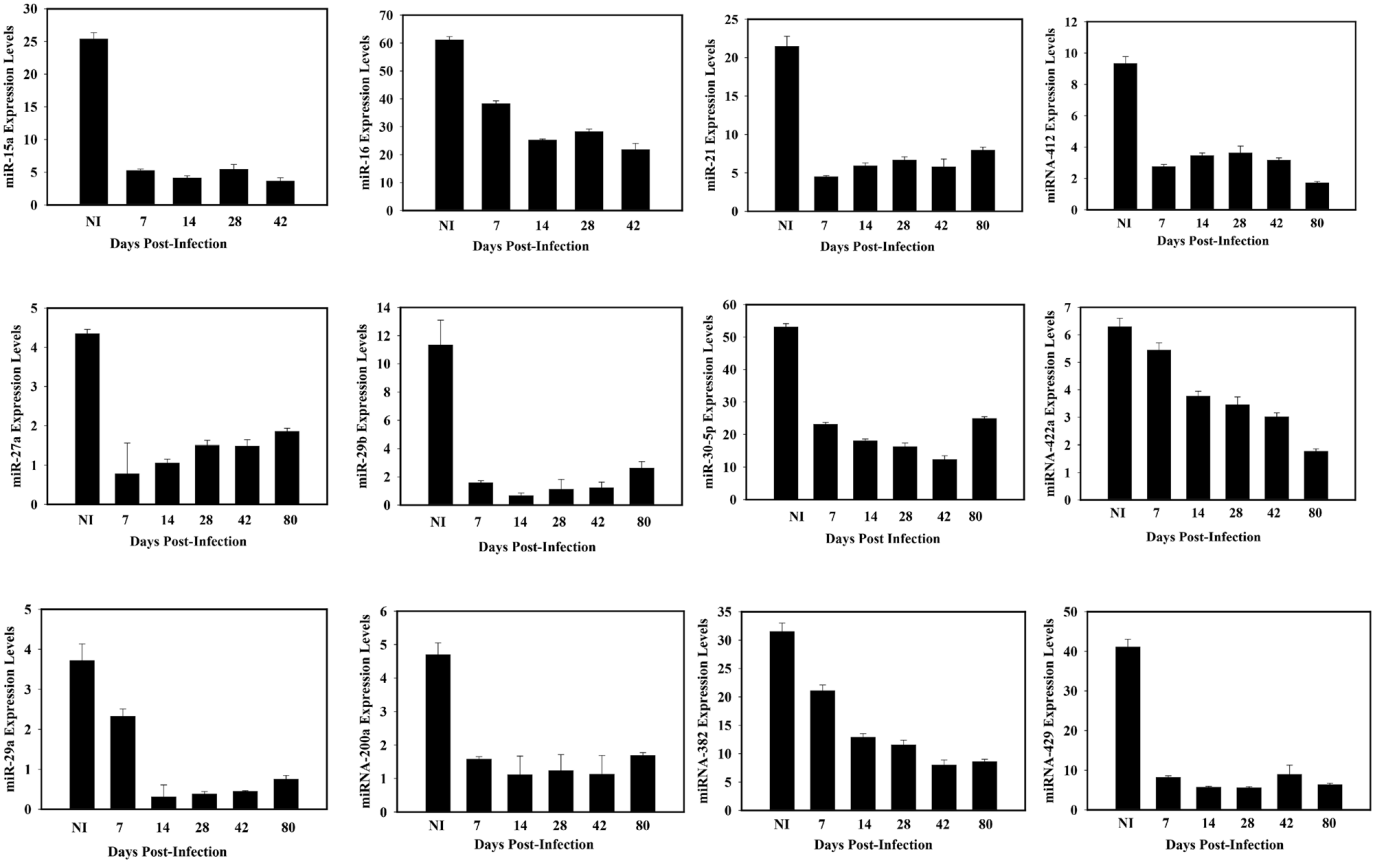
Chlamydial genital infection caused a sustained alteration of key miRNAs that control the functional integrity of epithelial cells (down-regulated miRNAs). Analysis of the pattern of expressed miRNAs in the oviducts of infected infertile mice and non-infected mice employed a combination of microarray and quantitative real time PCR as per Signosis’ miRNA Array IV service (Signosis, Inc. Sunnyvale, CA), as described in the Materials and Methods section. The labeled oligonucleotide probe mixture corresponded to 540 randomly selected miRNAs which have been reported to play a role in inflammation, apoptosis and cancer by literature search [101]. Quantitative Real-Time PCR was used to validate the microarray data using miRNA-specific oligo mix. Information about miRNA target mRNA genes was obtained from several Web-accessible miRNA database searching programs, including http://www.microrna.org, http://www.miRBase.org, http://www.targetscan.org and published reports [102]. Results presented in Table 1 **(are shown as Fig 1 Down-regulated**) were derived from oviducts harvested from 1 wk through at 80 days (11.4 wk) post-infection. Experiments were repeated 4 times with at least 6 oviducts in each group. Statistically significant changes had *P value* from ≤ 0.05.

**Fig 2:**
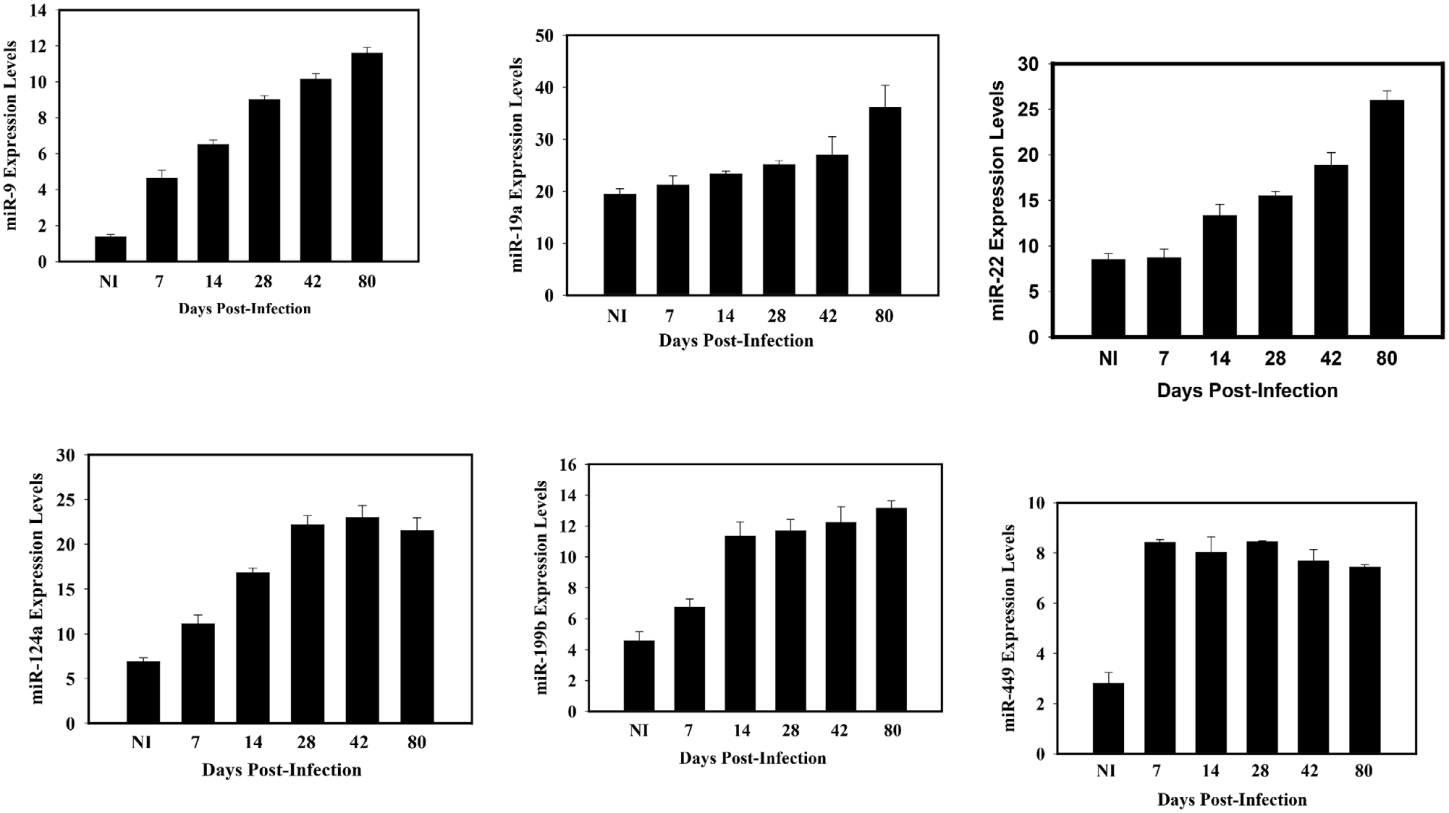
Chlamydial genital infection caused a sustained alteration of key miRNAs that control the functional integrity of epithelial cells (up-regulated miRNAs). Results were obtained as described in Fig 1 and selected miRNAs that were up-regulated have been presented.

From the functional analysis of miRNAs in Table 1, we have presented in Figs 1 and 2 (Fig 1: Down—regulated; Fig 2: Up-regulated) the detailed expression profiles of selected differentially expressed miRNAs whose altered expressions were sustained over the indicated duration of time after infection, and are known to regulate epithelial functional integrity relating to reproduction, fibrosis or tumorigenesis. Among the down-regulated miRNAs, both miR-15a and miR-16 control genes involved in epithelial cell viability, integrity and function, as well as cell cycle progression and angiogenesis. Also, both miRNAs act as tumor suppressors, inhibiting the expression of proteins involved in epithelial-mesenchyme transition (EMT) and tumorigenesis, especially the transcription factor Sox5 and Bcl2 [17,18]. The miR-29 family is required to preserve epithelial tissues from fibrosis via targeting for suppression components of extracellular matrix (ECM), especially collagen-I and -IV [8,19,20]; The suppression of the miR-29a and miR-29b is associated with the induction EMT, and it is a major trigger for cardiac and peritoneal fibrosis [20,21]; and so the suppression of members of miR-29 family during genital chlamydial infection is an indication of onset of fibrosis in the oviducts, which is a common pathologic consequence of genital chlamydial infection [2]. In addition, members of miR-29 family control cell invasion by targeting for suppression molecules that are involved in cell invasion and migration in the focal adhesion signaling pathway [22]; furthermore, miR-29a targets the heat-shock protein-47 (HSP47), a member of the serpin superfamily of serine proteinase inhibitors and a molecular chaperone involved in the maturation of collagen molecules; HSP47 is upregulated in fibrosis and several cancers especially cervical intraepithelial neoplasia where miR-29a is often down-regulated [23]. The miR-29s thus act as fibrosis and tumor suppressors, and their down-regulation, as in the reproductive system during genital chlamydial infection, leads to increased fibrosis and tumorigenesis. In addition, miR-203 and miR-204 which regulate EMT were down-regulated by chlamydia to enhance EMT. The down-regulated miRNA-382 and -409-5p are involved in controlling epithelial integrity and functions [15]. Furthermore, chlamydial infection caused a sustained suppression of key members of miR-200 family (miR-200a, -200b, -200c and -429) that maintain epithelial integrity phenotype by preventing EMT through downregulation of EMT-promoting transcription factors, especially the zinc finger E-box binding homeobox proteins ZEB1, ZEB2 and SIP1 proteins, [15,24]. In particular, miRNA-429 targets ZEB1, a transcriptional repressors of E-cadherin and other epithelial markers, and contraction-associated genes [15,24,25]. Thus, suppression of miR-429 releases its suppressive effect on ZEB1 and other pro-EMT transcription factors, thereby activating EMT that affects epithelial integrity, and promoting epithelial tumorigenesis. So miR-429 suppression is a major route for EMT induction and epithelial tumor promotion, which may be prominent in the genital epithelium during genital chlamydial infection.

Among the upregulated miRNAs during infection (Fig 2), miR-9 induces EMT by directly targeting the mRNA encoding E-cadherin [8]; its ectopic expression induced EMT in human mammary epithelial cells, and a sponge-trapping miR-9 consisting of multiple copies of a specific sequence complementary to miR-9 caused a reduction of invasiveness of a breast cancer cell line, certifying miR-9 as an EMT inducer and oncogenic miRNA [26,27]. The upregulated miR-19a affects epithelial integrity by regulating angiogenesis, epithelial differentiation, cell signaling through NF-kB, and cell proliferation. Also upregulated is the oncogenic miR-22 that triggers EMT, inhibits the ten-eleven-translocation gene 2 (TET2) tumor suppressors, causing an enhanced hematopoietic stem cell self-renewal, transformation and metastasis [28,29]. Finally, the upregulated miR-451 promotes cell migration and tumorigenesis. The results indicated that chlamydia infection altered the expression of miRNAs that control epithelial functional integrity and EMT, suggesting that chlamydia may induce EMT and the pathophysiological processes, including fibrosis, luminal and glandular epithelial tissue dysfunction and tumor promotion.

### *Chlamydia* infection of reproductive epithelial cells induces epithelial-mesenchyme transition (EMT)

**Fig 3:**
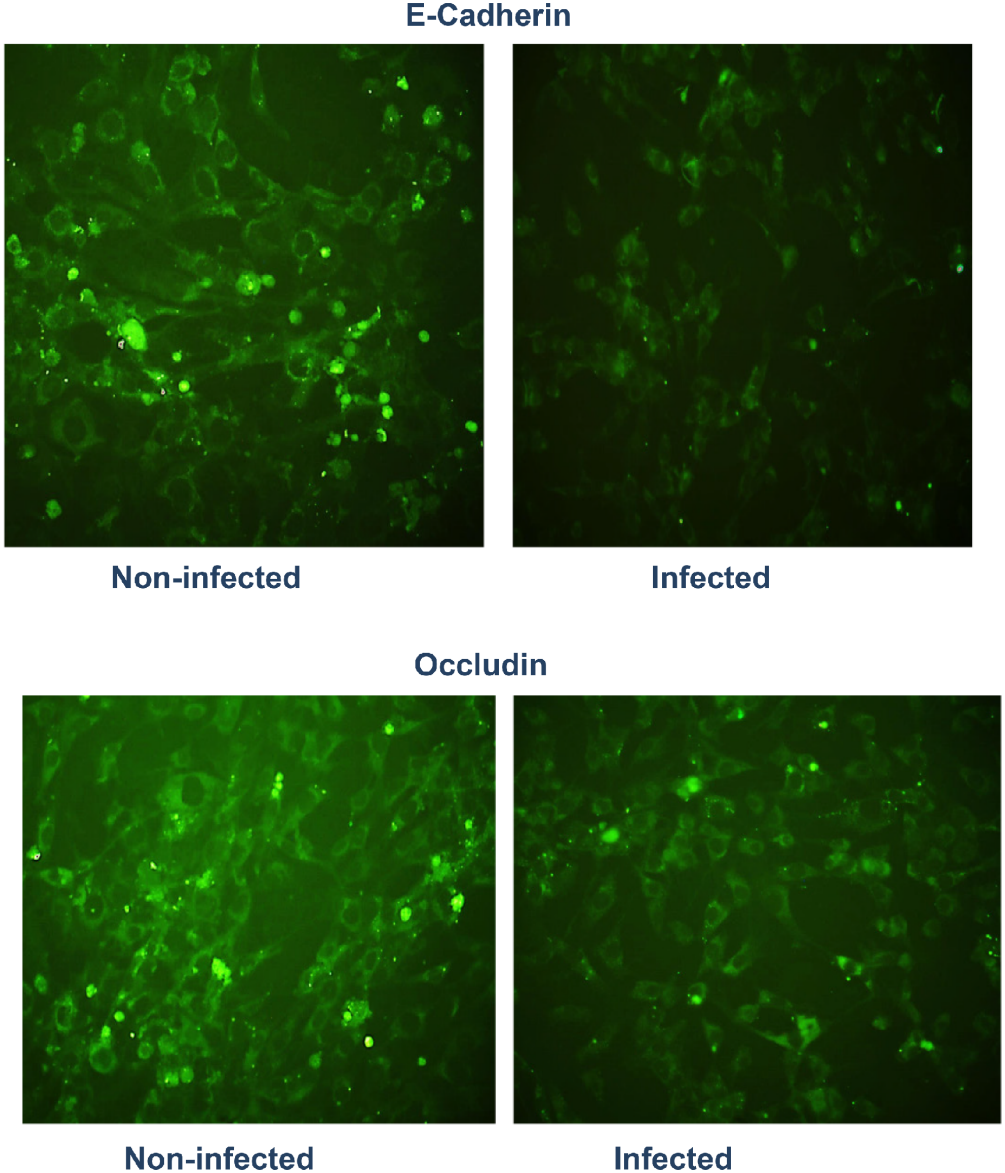
*Chlamydia* infection of reproductive epithelial cells induces epithelial-mesenchyme transition (EMT) (Loss of epithelial markers). Monolayers of murine oviduct epithelial cells were infected with *C*. *trachomatis* L and after 72h immunofluorescence staining of the cells for epithelial markers was performed on infected and non-infected monolayers by standard procedures. Fluoresceinated antibodies against the following markers were used: E—cadherin and occlusin. Antibodies stained both intracellular and surface antigens. Images were acquired at 20X objective on a Nikon fluorescent microscope with the same microscope settings and exposure time. The representative slides are from several repeated experiments showing identical results.

**Fig 4:**
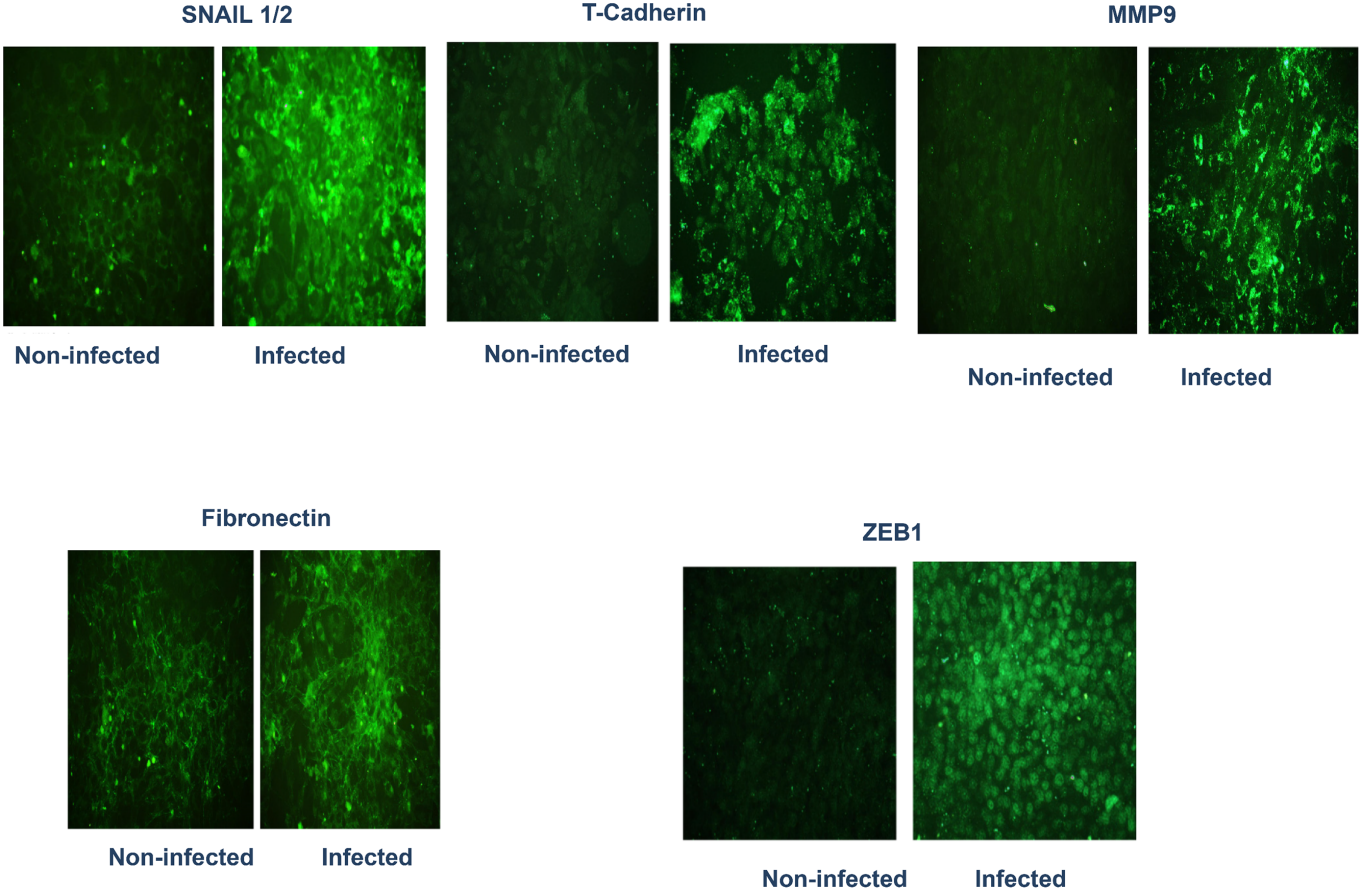
*Chlamydia* infection of reproductive epithelial cells induces epithelial-mesenchyme transition (EMT) (mesenchymal markers). Monolayers of murine oviduct epithelial cells were infected with *C*. *trachomatis* L and after 72h immunofluorescence staining of the cells for EMT markers was performed on infected and non-infected monolayers by standard procedures. Fluoresceinated antibodies against the following markers were used: T-cadherin, Snail1/2, fibronection, Zeb1 and MMP9. Antibodies stained both intracellular and surface antigens. Images were acquired at 20X objective on a Nikon fluorescent microscope with the same microscope settings and exposure time. The representative slides are from several repeated experiments showing identical results.

**Fig 5:**
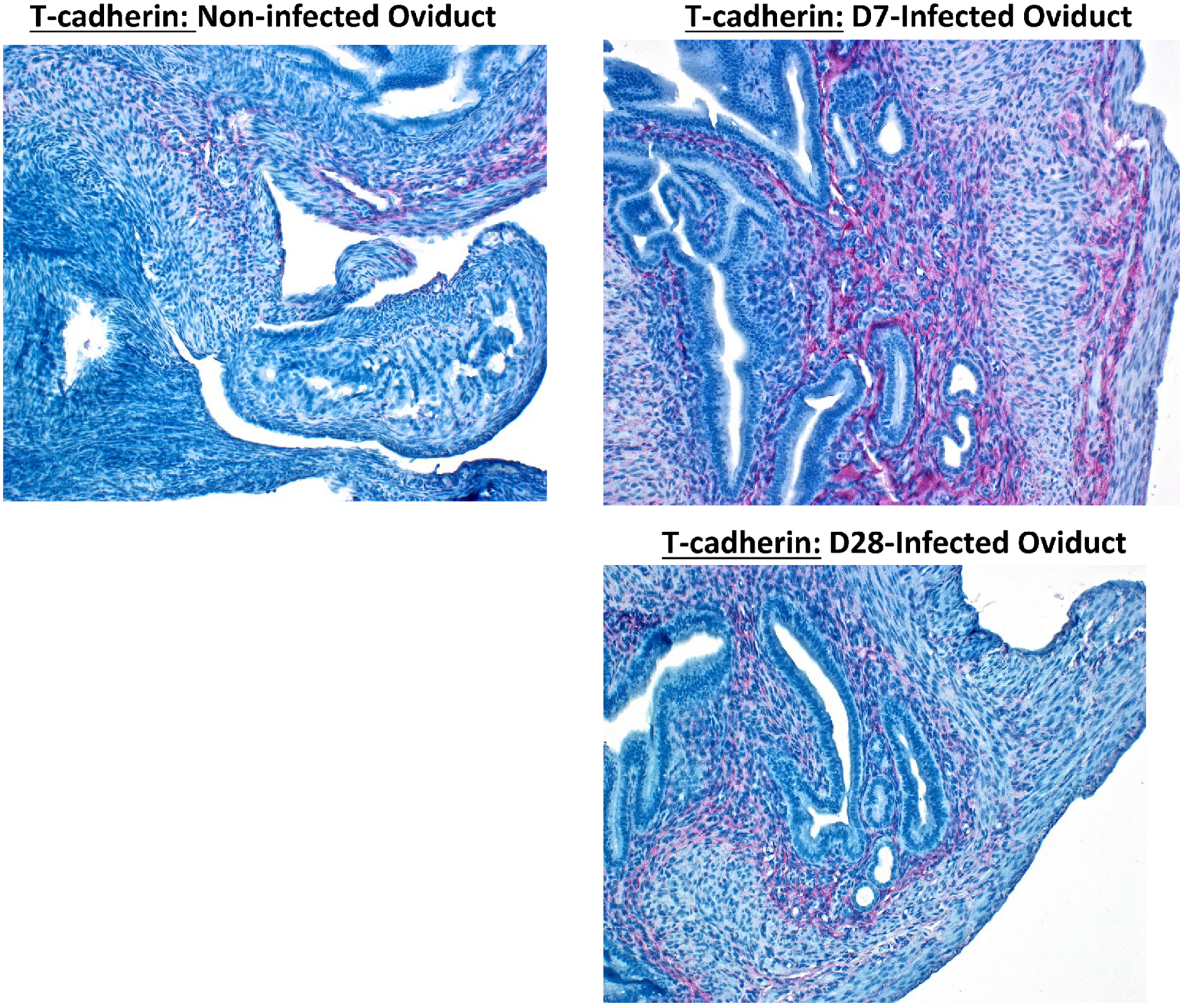
*In vivo* evidence of EMT after genital chlamydial infection. Immunohistochemistry (IHC) assays on sections of oviduct tissues from infected and non-infected mice were performed by using a polymer-based colorimetric indirect immunoalkaline phosphatase method with a polyclonal rabbit anti-T-cadherin antibody, as previously described [100]. Images were acquired at 20X objective on a Nikon fluorescent microscope. The representative slides are from several repeated experiments showing identical results.

We investigated whether chlamydial infection of isolated reproductive epithelial cells can induce EMT by altering epithelial characteristics and functions marked by suppression of E-cadherin and other epithelial markers with concomitant upregulation of mesenchymal markers. Results presented Figs 3 and 4 indicate that chlamydial infection of primary reproductive epithelial cells caused the downregulation of markers associated with normal epithelial integrity (E-cadherin and Occludin) (panel 2A) and the upregulation of mesenchymal markers (Snail1/2, Fibronectin, MMP9, T-Cadherin and ZEB1)(panel 2B) as an indication of EMT induction. So, chlamydia induces EMT, a major pathophysiological process associated with tissue fibrosis, loss of epithelial function and tumor invasion and metastasis [8,30]. Evidence for chlamydial induction of EMT *in vivo* was demonstrate by immunohistochemical staining of reproductive tract tissues from infected mice to identify mesenchymal markers. Thus, when immunohistochemistry (IHC) assays were performed on sections of oviduct tissues from infected and non-infected mice, it was found that immunostaining of T-cadherin, a marker of mesenchymal phenotype was upregulated in the oviducts from infected mice mainly in the lamina propria and interstitial connective tissues (Fig 5). The dramatic increase in T-cadherin in infected oviducts was greater on day 7 post-infection compared to day 28 post-infection oviducts. Although a clear difference is yet to be observed in the expression of E-cadherin between infected and non-infected oviducts, the results indicated that there was upregulation of at least a reliable mesenchymal marker in the genital epithelium after genital chlamydial infection, indicating that *Chlamydia* also induced EMT *in vivo*.

### *Chlamydia* infection caused the altered expression of proteins that regulate epithelial functional integrity, EMT and tumorigenesis in the reproductive system

**Fig 6:**
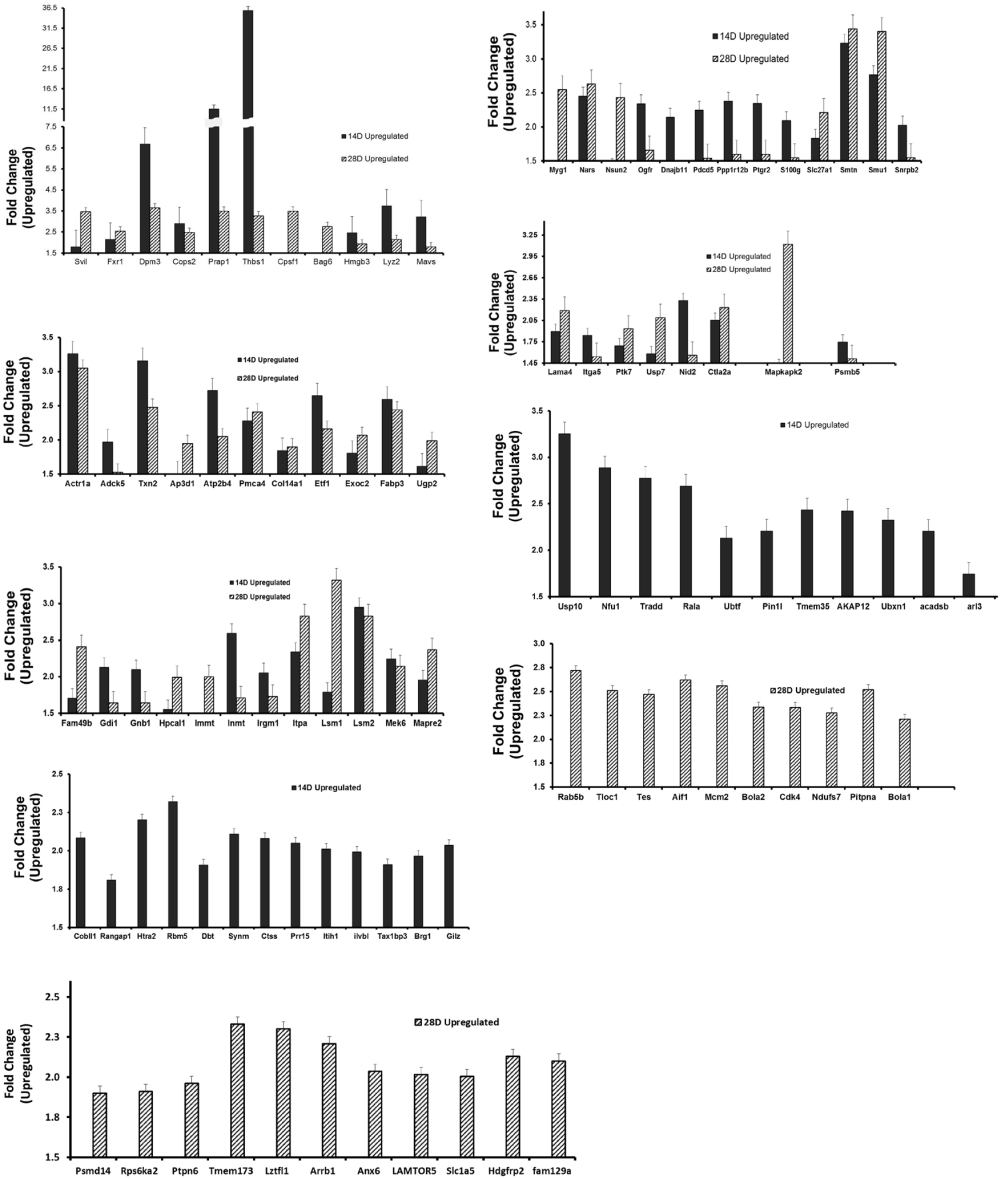
*Chlamydia* infection caused the altered expression of proteins that regulate epithelial functional integrity and pathologic EMT in the reproductive system (up-regulation). The quantitative differential proteomics with isobaric tags for relative quantitation (iTRAQ) labeling and LC-MS analysis was used to identify proteins that were differentially expressed in the oviducts from chlamydial infected mice, as previously described [103,104]. Briefly, trypsinized proteins extracts were labeled using the 114, 115, 116 and 117 iTRAQ tags and analyzed by MS, as described in the materials and Methods section. Protein/peptide identification and quantification were performed with the Protein Pilot Software version 4.5 and MASCOT, including the search of the database for murine or human proteins (http://www.uniprot.org). Fold Change represented protein expression in infected oviducts divided by protein expression in uninfected oviducts. The down-regulated molecules were expressed in percentage to avoid negative values. Only protein identified by at least 2 peptides and with a P-value < 0.05 were used for quantitation. The biological and functional properties of the proteins and their physiological and disease pathways were established through http://www.elsevier.com/online-tools/pathway-studio (Pathway studio 10; www.elsevier.com/online-tools/pathway-studio) and other specialized biomedical databases that include PubMed and Medline searches. **Results show** molecules upregulated at D14 and D28 p.i.

We hypothesized that chlamydia will alter the expression of proteins that regulate epithelial integrity, physiologic functions and pathologic EMT in the reproductive system, and several of these proteins will be targets of miRNAs that control epithelial integrity through EMT. Using a quantitative iTRAQ and LC-MS analysis, we identified proteins that were differentially expressed (either up- or down-regulated) in the oviducts from chlamydial infected mice, as compared to uninfected mice. Fig 6 presents a list of upregulated proteins. Notable among these proteins are: PRAP1 (proline-rich acidic protein 1), a hormonally regulated, luminal epithelium-specific protein that is a marker for successful embryo implantation because it is expressed when the embryo has been situated at the implantation site [31]. The up-regulation of PRAP1 in the oviduct following genital chlamydial infection (3- to 11-fold between 2 and 4 wk post-infection) is prognostic for premature implantation in the oviduct that causes ectopic pregnancy. *Chlamydia* also dramatically upregulated the potent EMT-inducing and fibrogenic protein, thrombospondin 1(Thbs1 or Tsp1) by 3- to 31-fold in 2–4 wk p.i. The Bcl-2-associated athanogene 6 BAG6 (also called BAT3), encoded within the class III region of the human MHC is involved in the ubiquitin-dependent elimination of defective, mis-localized, misfolded and aggregation-prone proteins that prevents aggregated misfolded protein diseases, as well as in cell survival, membrane protein biogenesis and apoptosis regulation [32]. The upregulated high mobility group box 3 (HMGB3) protein is a member of a family of proteins containing one or more high mobility group DNA-binding motifs and plays an important role in DNA replication, recombination, and repair; HMGB3 is under the control of tumor suppressive miRNAs such as miR-205 and is overexpressed in a variety of human cancers where those miRNAs are suppressed, and proposed to regulate cancer stemness [33,34]. Supervillin (Svil) [35,36] is a peripheral membrane protein that binds F-actin and myosin II, reorganizes the actin cytoskeleton and potentiates invadopodial function, thereby facilitating cell motility; Svil suppresses the tumor suppressor p53, prevents apoptotic cell death by enhancing cell survival, and it therefore plays a role in tumorigenesis. The suppressor of mec-8 and unc-52 homolog protein (Smu1) is a WD40-repeat protein encoded by an evolutionarily conserved gene found in most eukaryotic organisms and it is important for regulating DNA replication and cell growth [37]. The Sm-like protein, Lsm1 is an oncogene [38,39] that forms part of a cytoplasmic protein complex, Lsm1-7-Pat1, which is involved in mRNA degradation, turnover and control of gene expression through the 5'-to-3' mRNA decay pathway, linking deadenylation to decapping. It regulates histones mRNAs to ensure genomic stability. Lsm1 is over-expressed in multiple tumor types, including over 80% of pancreatic tumors, and increased levels of Lsm1 protein have been shown to induce carcinogenic effects [39]. The fragile-X-mental retardation-related protein 1 (FXR1) is an RNA-binding protein [40] that regulates the translation of key cytoskeletal components in myoblasts and therefore critical for muscle development; and its co-regulation with miR-29 suggested it plays a role in fibrosis [19]. PSMD14 and PSMB5 are components of the 19S and 20S proteasome, respectively that are involved in the deubiquitination during proteasomal degradation and cell cycle events that ensure cell growth, survival, and stemness that prevents senescence [41]. COP9 signalosome subunit 2 (Cops2) is one of the 8 subunits of COP9 or CSN (CSN1-CSN8) [42,43] The COP9 signalosome (CSN) is an evolutionarily conserved protein complex with homologies to the 19S lid subcomplex of the 26S proteasome. COP9 play a role in regulating the degradation of polyubiquitinated proteins including tumor suppressors and cell cycle proteins involved in cell proliferation, senescence, EMT and tumorigenesis [44]. Actr1a is a member of the actin-related proteins (ARP) with cytoskeletal function relating to cell migration and membrane transport; it has altered expression in certain cancers [45]. Txn2 is thioredoxin 2 (Trx2), a key mitochondrial protein that regulates cellular redox and survival by suppression of mitochondrial reactive oxygen species generation and inhibition of apoptosis stress kinase-1 (ASK1)-dependent apoptotic signaling [46]. Dpm3 is the third subunit of the dolichol-phosphate-mannose (DPM) synthase complex located on the endoplasmic reticulum and required for protein gycosylation modification [47]. The upregulation of Dpm3 may indicate heightened ER activity and possibly ER stress. The fatty acid-binding protein-3 (FABP3) is important for fatty acid transport and play a role in cell survival [48]. Thus, these results validated the hypothesis that genital chlamydial infection will up-regulate the expression of proteins that promote pathogenic EMT, fibrosis, epithelial dysfunction, and tumor promotion in the reproductive system, and several of the proteins are targets of miRNAs that control epithelial integrity EMT.

**Fig 7:**
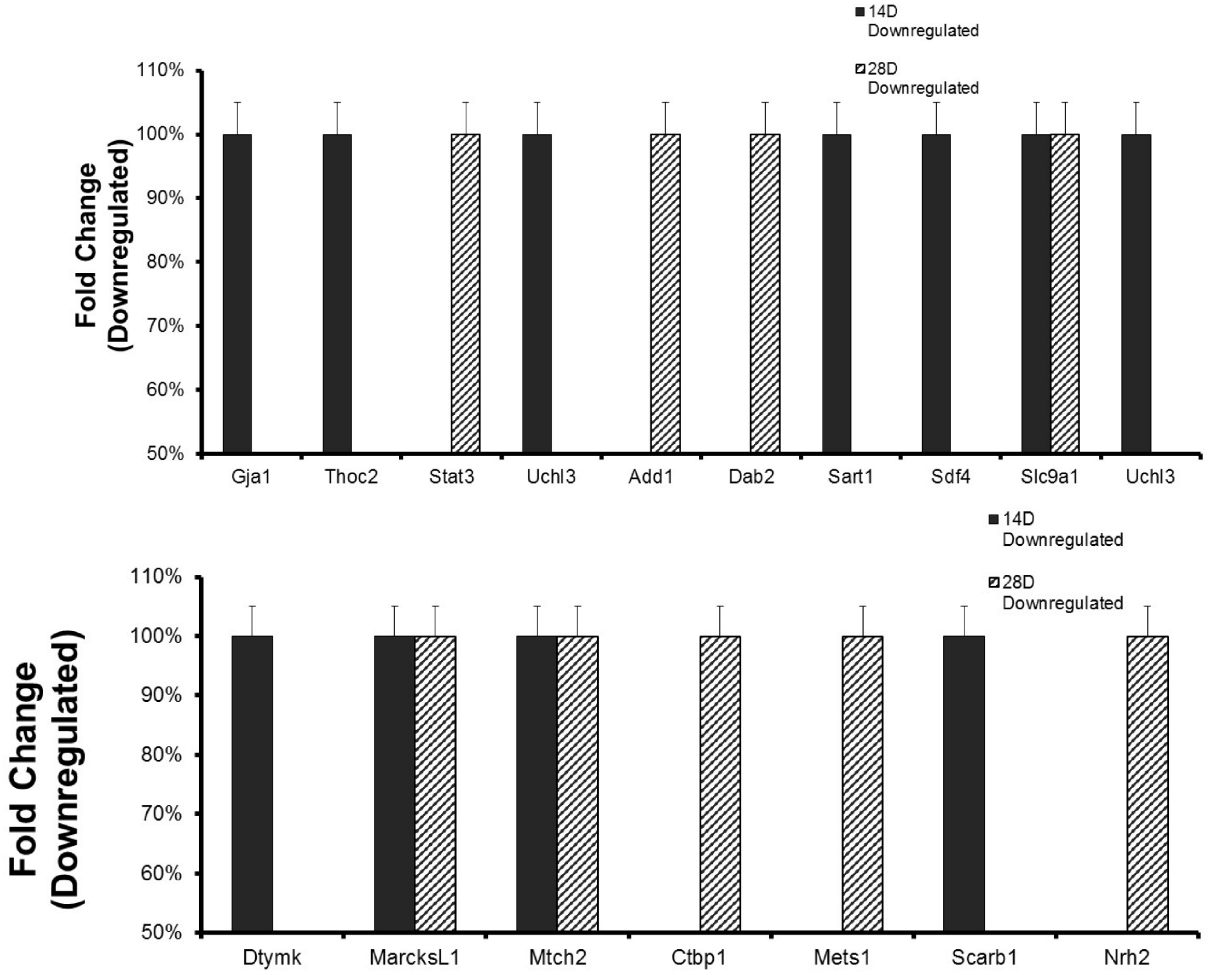
*Chlamydia* infection caused the altered expression of proteins that regulate epithelial functional integrity and pathologic EMT in the reproductive system (Down-regulation). Results were obtained as described in Fig 6 and selected molecules that were down-regulated have been presented.

Fig 7 presents a list of down-regulated proteins in the oviducts from chlamydial-infected mice, as compared to expression levels in oviducts from sham/non-infected animals. Notable among the down-regulated proteins that are at least 50% lower than normal expression in the absence of infection are: The gap junction a-1 protein (Gja1), also called the major gap junction protein Connexin 43 (Cx43), is a contractility-associated protein that is markedly enhanced in response to estrogen in the reproductive tract epithelial stromal cells and the embryo during the early phases of pregnancy. It plays a vital role in oviduct contraction for embryonic transport, implantation and parturition [49–51]. The down-regulation of Cx43 in the oviduct during chlamydial infection indicates a decreased tubal contractility that may cause ectopic pregnancy. Gap junction-targeting has also become a pathogenic mechanism of certain infectious agents, including reproductive system diseases such as endometritis and inflammatory CNS diseases [51,52]. A STAT3-signaling mediated regulation of levels of IL-6 is required for successful pregnancy [53] and the IL-6/STAT3 signaling pathway has anti-senescence and growth-promoting functions in human tumors [54]. STAT3 signaling involving the leukemia inhibitory factor (LIF) is critical for embryo implantation and reproductive fertility, and LIF deficiency is associated with unexplained recurrent abortion and infertility in women and inactivation of STAT3 causes pregnancy failure [13,55]. The down-regulation of STAT3 in chlamydia-infected infertile mice therefore predicts pregnancy failure and infertility observed in these mice. UCHL3 is ubiquitin carboxyl-terminal esterase L3 or ubiquitin thioesterase or ubiquitin hydrolase, one of the deubiquitinating enzymes (DUBs) that control proteolysis by reversing ubiquitination in the regulation of the ubiquitin-proteasome protein degradation system (UPPDS); the aberrant reduced or blocked expression of UCHL3 compromised epithelial functional integrity by reducing epithelial sodium channel [56] and affects fertilization and pre-implantation embryonic development in vivo [57]. Add1 is a component of adducin (ADD), a heterodimeric actin-binding cytoskeleton protein consisting of an *α*-subunit with either a beta- or a gamma-subunit where the alpha-subunit (Add1) is known to increase renal sodium reabsorption and therefore considered to be involved in the pathophysiology of essential hypertension [58] possibly through regulation of epithelial sodium channel. Its downregulation in chlamydial infected infertile animals would suggest an alteration in epithelial functional integrity. Disabled-2 (Dab2) is an epithelial polarity-determining protein crucial for epithelial cell apico-basolateral orientation and epithelial organization during development, and as an endocytic adaptor protein with a major role in clathrin-mediated endocytosis and trafficking and recycling of membrane proteins [59,60]. Dab2 is also considered a tumor suppressor and lost in several malignant cancers [61]. The loss of Dab2 during chlamydial infection will favor EMT, loss of epithelial polarity and function, and tumor promotion. The C-terminal-binding protein 1 (Ctbp1) is a transcriptional corepressor that may play a role in controlling EMT and fibrosis by suppressing MMP9 [62], apoptosis and cell proliferation [63]. Mets1 is mesenchyme-to-epithelium transition (reverse EMT) protein with SH3 domains 1 (also known as CD2-associated protein (CD2ap) [64,65], acting as an adapter protein between membrane proteins and the actin cytoskeleton; it plays a role in receptor clustering and cytoskeletal and apico-basolateral polarity; deficiency of CD2ap causes defects in epithelial foot processes, accompanied by mesangial cell hyperplasia and ECM deposition. Down-regulation of Mets1 during chlamydial infection would favor EMT, fibrosis and compromised epithelial functional integrity. CD2ap is a target of miR-199 regulation. The mitochondrial carrier homolog-2 (Mtch2) protein regulates membrane permeability and apoptosis and its suppression by miR-135b promotes certain carcinomas [66,67]. Myristoylated alanine-rich C kinase substrate-like 1 (MARCKSL1) is a tumor suppressor that regulates actin cytoskeleton, cell movement, EMT, fibrosis and the invasiveness and metastasis of tumors by suppressing lysyl oxidase-like 2 (LOXL2) and the FAK/Akt/mTOR signaling pathways [68] The down-regulation of MARCKSL1 by chlamydia promote EMT, fibrosis and tumorigenesis. Deoxythymidylate kinase (thymidylate kinase, Dtymk) is important for cellular DNA synthesis and appears to act with the tumor suppressor *LKB1/STK11* to coordinates cell growth, polarity, motility, and metabolism [69]. SART1 is a spliceosome factor involved in RNA splicing and pre-mRNA processing, and a regulator of IFN's antiviral effects [70]. Sdf4 is a member of the stromal cell derived factor (SDFs) family and functionally classified as a chemokine; SDF-2, SDF-4 and SDF-5 are expressed in mammary tumor tissues and cells and a reduced level of SDF-2, SDF2-L1 and SDF-4 are associated with a poor clinical outcome [71]. Sodium/hydrogen exchanger 1 (Slc9a1 or NHE-1) is regulator of membrane permeability, sodium ion transport, apoptosis, cell differentiation and growth; it is suppressed during ER stress that causes epithelial injury [72]. The down-regulation of NHE-1 during chlamydial infection would indicate the induction of ER stress. Scarb1 encodes the scavenger receptor class B type 1 (SR-B1) that is important for female hormonal synthesis, steroidogenesis and fertility; its knock down led to reproductive infertility [73,74]. Thus, its down-regulation during genital chlamydial infection would suggest that the mice will be infertile. The death domain-containing membrane protein, neurotrophin receptor associated death domain (NRADD), also known as Neurotrophin receptor-alike death domain protein (Nrh2) promotes apoptosis through [75]. Its down-regulation is anti-apoptotic, which would promote cell survival and tumorigenesis. These results confirm the converse hypothesis that genital chlamydial infection will down-regulate proteins that promote reproductive fertility, normal epithelial function or that control pathologic EMT.

### The role of T cell-derived TNF-alpha and caspase activation in chlamydial-induced infertility and EMT

**Fig 8:**
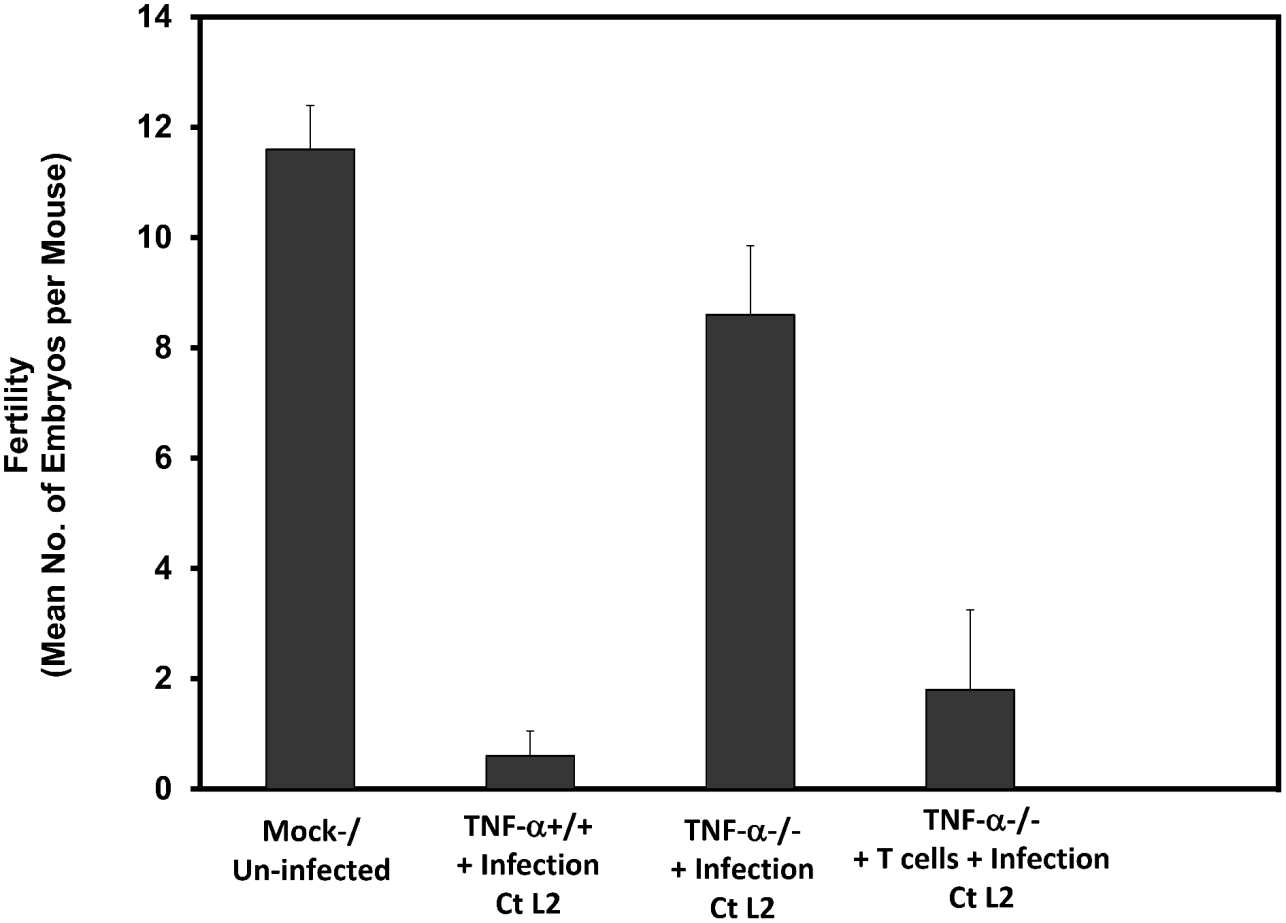
Role of T cell-derived TNF-alpha in chlamydial-induced infertility. Groups of female mice (TNF-alpha^-/-^, controls TNF-alpha^+/+^, and TNF-alpha^-/-^ that received 10^7^ purified T cells from TNF-alpha^++^ mice) were infected intravaginally with *C*. *trachomatis* L2 (WT-L2), as described in the Materials and Methods section. Infected and noninfected control mice were mated and scored for pregnancy and no. of pups. The plotted data are the means (SEM) for groups from 2 independent experiments with 6 mice per experimental group.

**Fig 9:**
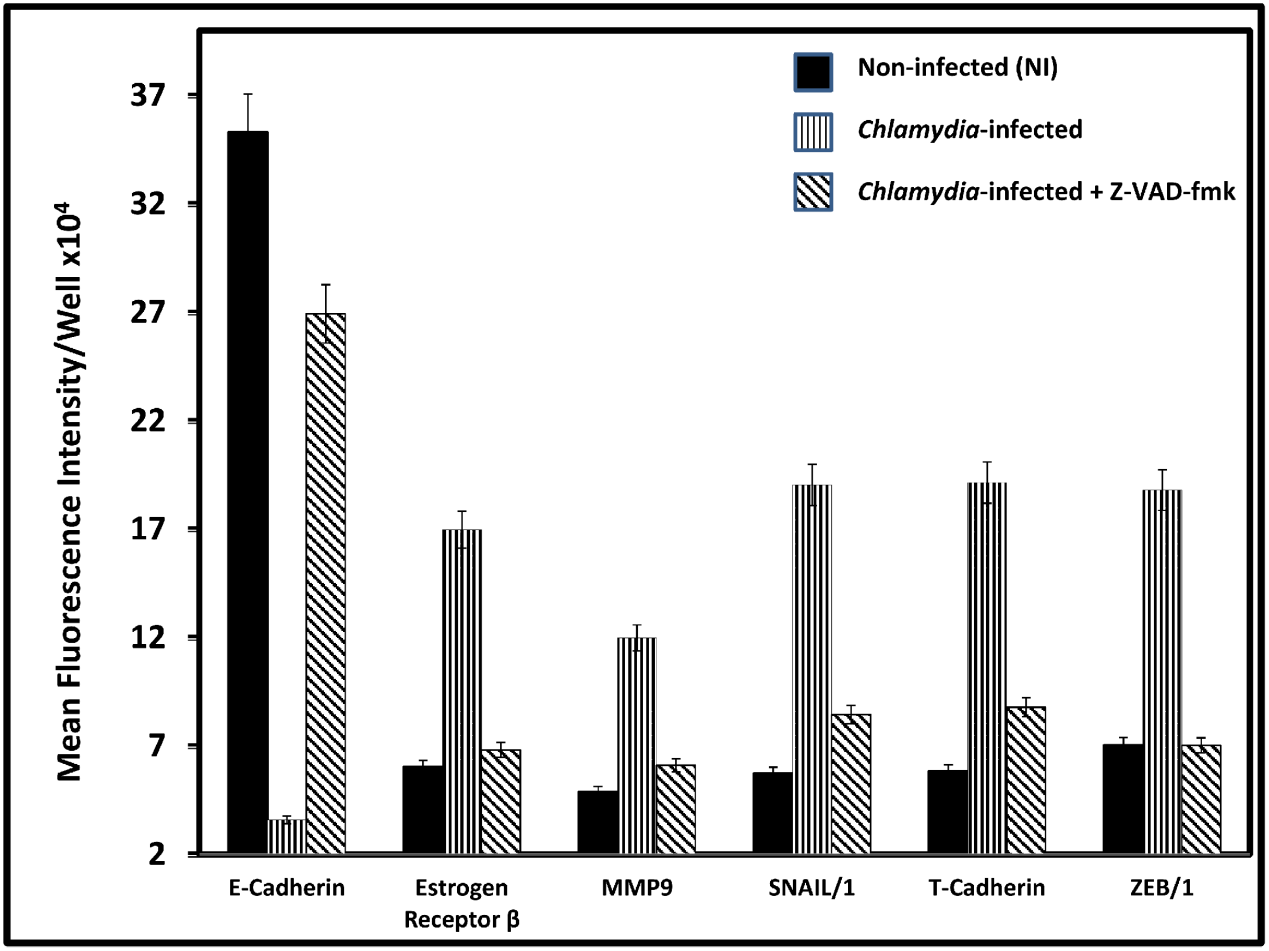
Caspase inhibition prevented chlamydial-induced EMT. Monolayers of murine oviduct epithelial cells were infected with *C*. *trachomatis* L and after 72h immunofluorescence staining of the cells for epithelial and EMT markers was performed on infected and non-infected monolayers by standard procedures. Z-VAD-FMK and the control Z-FA-FMK were used in culture at 50 micromole. Quantification of fluorescence was performed by scanning fluorescent-stained cells at 20X objective on a Nikon fluorescent microscope using the NIS-Elements Imaging Software version 3.20 (Nikon Instruments Inc, Melville, NY USA). Images were acquired for experimental and control cultures with the same microscope settings, exposure time and background. The mean fluorescence intensity and standard deviations of 6 out of 10 scanned fields per slide were calculated automatically by the software. Plotted data were derived from 5 independent experiments.

The requirement for T cells, the T cell-derived cytokine TNF-alpha and caspase-3 for chlamydial-induced infertility and tubal pathologies [3,76–78] suggested that the pathogenesis of chlamydial reproductive complications involved TNF-alpha signaling and caspase activation. Thus, the corroborative results presented in Fig 8 indicates that, although TNF-alpha ^-/-^ mice are resistant to chlamydia-induced infertility, the adoptive transfer of T cells from wild-type (TNF-alpha^+/+^) mice into knockout (KO) mice restored infertility in infected mice. Since TNF-alpha is a potent EMT inducer [7], recent results showing that caspase activation by chlamydia caused the cleavage inactivation of dicer, a key enzyme in miRNA biosynthesis [3], suggested that alteration in dicer activity led to changes in expression of miRNAs that drive EMT and the pathologic consequences. In fact, female animals with targeted knockout of dicer from the reproductive tissues exhibited miRNA dysregulation, infertility and oviduct pathologies similar to tubal factor infertility in humans [79]. And the local application of caspase inhibitors prevented chlamydia-induced infertility [3]. Therefore, we reasoned that, if caspase activation, Dicer inactivation and miRNA dysregulation are the drivers of EMT that causes *Chlamydia*-induced reproductive pathologies, EMT should be sensitive to caspase inhibition as well. Thus, as a mechanistic investigation of the role of caspase activation in EMT induction, and the possibility of pharmacological intervention, we tested the hypothesis that caspase inhibition would prevent chlamydial-induced EMT. As shown in Fig 9, treatment of infected epithelial cells with the pan-caspase inhibitor Z-VAD-fmk prevented EMT, marked by retention of normal epithelial E-cadherin and suppression of mesenchymal MMP9, SNAIL1/2, T-cadherin, ZEB1 and estrogen receptor-beta markers and regulators. Perhaps this is the first report of EMT inhibition by an anti-caspase agent, which opens up a new translational approach to therapeutic targeting of EMT-driven diseases such as fibrosis and tumor promotion, requiring safe, non-toxic and human compatible caspase inhibitors.

## Discussion

We investigated the molecular pathogenesis of the complications of genital chlamydial infection and as a co-factor in HPV-associated cervical epithelial carcinoma. We tested the hypothesis that chlamydia induces pathologic EMT that alters fertility-related epithelial functions, promotes fibrosis and tumorigenesis. The results so far have revealed that chlamydia induces EMT, involving the activation of T cells, TNF-alpha signaling, the caspases, dicer inactivation, the disruption of crucial miRNAs and proteins that regulate fertility and epithelial functional integrity, and promotes fibrosis and tumorigenesis in the reproductive system [9]. Among the down-regulated miRNAs is the miR-29 family that protects epithelial tissues from fibrosis [8,19,20]. EMT promotes fibrosis by converting resident epithelial cells into apoptosis-resistant myofibroblasts that deposit ECM proteins; and unlike in wound healing, the cells persist in ECM causing a hypertrophic scar or keloid on tissues or fibrosis in organs, compromising organ function, and sometimes causing organ failure [9]. The suppression of the miR-29a and miR-29b induces EMT, and culminated in cardiac, kidney, pulmonary and peritoneal fibrosis but could be ameliorated with miR-29 gene therapy [20,21]. Thus, the suppression of members of miR-29 family following genital chlamydial infection is an indication of onset of EMT and fibrosis in the reproductive system, which is a common pathologic consequence [2]. Perhaps this is the first reported finding of the suppression of miR-29b as a likely cause of tubal fibrosis during genital chlamydial infection, suggesting that chlamydia-induced fibrosis may benefit from a miR-29-based gene therapy in future. In addition, members of miR-29 family control cell invasion by suppressing molecules involved in cell invasion and migration in the focal adhesion signaling pathway, including laminin gamma1 (LAMC1), laminin gamma1 (LAMC2) and alpha6 integrin (ITGA6) [22]; and heat-shock protein-47 (HSP47), a target of miR-29a, is involved in the maturation of collagen molecules, and is upregulated in fibrosis and several cancers especially cervical intraepithelial neoplasia where miR-29a is often down-regulated [23]. Furthermore, miR-429 is an active inhibitor of the transcription factors that mediate EMT, including direct suppression of ZEB1 to maintain E-cadherin expression. Silencing the miR-200 family, especially miR-429, induces EMT and tumor promotion in several experimental and clinical practice systems [9]. In addition, miR-204 suppresses EMT by directly repressing TGF3R2 and SNAI2 (SLUG) to prevent the down-regulation of claudin junctional proteins and preserve cell membrane voltage and conductance that ensure epithelial physiology and barrier functions; and miR-203 suppresses EMT by promoting mesenchyme-to-epithelial transition (MET) [10]. On the other hand, among the up-regulated miRNAs, miR-9 induces EMT by its direct action on CDH1, the E-cadherin gene; and as an oncogenic miRNA, it is upregulated in several primary and in metastatic cancers [9]. MiR-22 promotes stemness and metastatic phenotype of cancer stem cells through suppression of the miR-200 family by increasing the methylation of their genes [80,81]. The persisting dysregulation of miRNAs after chlamydial infection implied that EMT and its pathophysiologic consequences have long-term effects.

The demonstration that *Chlamydia* infection can directly induce EMT *in vitro* and *in vivo*, marked by E-cadherin loss or a cadherin switching from E- to N- or T-cadherin is the prototypical marker of EMT [8]. E-cadherin is required to maintain epithelial functional integrity by its role in cell-cell adhesive interaction through its cytoskeletal interactions with cytoplasmic catenins that provide the epithelia cobble-stone-like tissue architecture, ensure epithelial barrier, and aggregate forms as functional tissue glands. Also, E-cadherin is critical for post-fertilization embryo-maternal interaction in the oviduct and uterus, for preimplantation embryonic survival, decidualization and implantation, and female fertility [13]. In fact, women with endometriosis show a remarkably down-regulated E-cadherin and beta-catenin expression in the epithelial cells of their mid-secretory endometrium [13]. On the other hand, T-cadherin expression promotes cell motility, invasiveness, migration and survival to enhance tumorigenesis [82]. Thus, chlamydia-induced EMT-associated alteration of cadherins in the luminal epithelium of the oviduct can cause reproductive abnormalities. More so, *Chlamydia* induced pro-EMT transcription factors and regulators, including the zinc finger E-box binding homeobox protein, ZEB1 and Snail1/2, thrombospondin1 (Thbs1 or Tsp1). Tsp1 is upregulated in several cancers especially those with invasive properties, such as endometrial cancer where its upregulation inversely correlated with miR-17-5p [83]; and its down-regulation by siRNA-mediated silencing or antibody neutralization reduced invasiveness and inhibited *in vivo* motility of cancer cells [84,85]. Other proteins upregulated by chlamydia in the reproductive system that contribute to EMT, fibrosis, oncogenesis and tumor progression, invasiveness and metastasis are BAG6 (also called BAT3) [32], the HMGB3 protein [33,34], Svil [35,36], Smu1 [37], the oncogene Lsm1 [38,39], FXR1 [40], PSMD14 and PSMB5 [41], Cops2 or CSN2, [42–44], Actr1a [45], Txn2 or Trx2 [46], Dpm3 [47], and FABP3 [48]. Several EMT inducers such as cytokines, hypoxia, bile acids and nicotine also promote tumorigenesis by enhancing invasive cell growth, local invasion and distant spread [9]. The role of chlamydia-inducible, cytokines such as TNF-alpha and TGF-beta in EMT and fibrosis has been well established [86]. Thus, the ability of estrogen to induce EMT involving miRNA alterations [87], and our results showing that chlamydia induced EMT and estrogen receptors, would suggest that chlamydia may induce EMT via the estrogen receptor-signaling pathway. Consistent with the miRNA profiles and up-regulation of pro-EMT proteins, our results further showed that the chlamydial down-regulated proteins are those that maintain epithelial integrity, support reproductive fertility, control fibrosis and act as tumor suppressors. Among these, the major gap junction a-1 protein (Gja1; or Connexin 43, Cx43), is required for oviduct contraction for embryonic transport to the uterus, implantation and parturition [49–51]. The down-regulation of Cx43 in the oviduct during chlamydial infection indicates a decreased in tubal contractility that may cause ectopic pregnancy and poor embryonic development. Other relevant down-regulated proteins are: STAT3, the leukemia inhibitory factor (LIF), ubiquitin carboxyl-terminal esterase L3 (UCHL3), Add1 (a component of adducin, ADD), disabled-2 (Dab2), and an epithelial polarity-determining protein crucial for epithelial cell apico-basolateral orientation and epithelial organization, Ctbp1. Also down-regulated are: Mets1 (a mesenchyme-to-epithelium transition protein), CD2-associated protein (CD2ap), the mitochondrial carrier homolog-2 (Mtch2), myristoylated alanine-rich C kinase substrate-like 1 (MARCKSL1) protein, lysyl oxidase-like 2 (LOXL2), deoxythymidylate kinase (thymidylate kinase, Dtymk), SART1, the stromal cell derived factor-4 (SDF4), sodium/hydrogen exchanger 1 (Slc9a1 or NHE-1), Scarb1 (the scavenger receptor class B type 1; SR-B1), and neurotrophin receptor associated death domain (NRADD or Nrh2). These molecules are required for either successful pregnancy, implantation, maintenance of epithelial integrity, or control of EMT and fibrosis, and tumor suppression [13,53–61,63–75,88–90].

The sensitivity of chlamydia-induced EMT and infertility to caspase inhibitors [3] confirmed that caspase inhibition of dicer represents a key upstream initiating event for miRNA-mediated, chlamydia-induced EMT; this finding validates the proposed link between caspase activation, miRNA disruption due to dicer inactivation, and EMT induction. Furthermore, clinico-pathologic studies indicate that the acquisition of invasive and metastatic characteristics by transformed cells involves the induction of EMT, the down-regulation of tumor suppressors such as p53, and the maintenance and expansion of cancer stem cells, CSCs. EMT inducers such as TNF-alpha signaling through both the canonical Smad and non-Smad (Akt and ERK1/2), Wnt and focal adhesion pathways [9] activate oncogenic TFs and miRNAs whose gene expression regulation cause the enhancement of cell migration and epithelial scattering characteristic of EMT [8]; and this drives a subset of transformed primary epithelioid cells into self-renewing tumor-initiating cells or CSCs, (CD44^hi^CD24^low^) that efficiently generate new tumors due to clonal expansion, motility and invasive state that enable them to initiate local invasion and distal metastasis [7,9]. Thus, EMT-inducing agents are co-factors in the development and progression of several cancers [9]. Our working hypothesis is that EMT-inducing agents, including cytokines, estrogen [87,91] and chlamydia, are co-factors for HPV-related invasive cervical carcinoma. Cervical cancer is the most common malignancy in females worldwide and a leading cause of mortality among gynecological cancers. Oncogenic types of HPV (e.g., 16 and 18) are important in the development of precursors of cervical cancer [92] but the fact that only a fraction of HPV-infected females develop the cervical carcinoma has prompted the search for co-factors in the progression to invasive cervical cancer [93]. Our results are consistent with the hypothesis that chlamydial induction of EMT during co-infection with HPV provides the pathophysiological basis for the cofactor in HPV-induced cervical carcinoma. An ongoing study is using the HPV 16 E6/7 transgenic mouse model that requires estrogen as co-factor for accelerated development of invasive cervical carcinoma [94,95], to determine whether chronic genital chlamydial infection can replace estrogen in disease pathogenesis. Besides, while evidence of EMT has been observed in the induction or progression of several tumors, our finding may represent the first evidence of a direct relationship between a co-factor and EMT. Thus, these findings provide a novel understanding of the molecular pathogenesis of chlamydia-induced infertility and its co-factor role in cervical carcinoma, which may guide to a rational strategy to prevent complications. Targeting EMT and especially the components of the signaling pathways of inducers with small molecule inhibitors such as antagomirs, siRNA- or anti-sense oligonucleotide, miRNA sponges, or antibodies and chemical inhibitors, represents an important therapeutic strategy for the clinical management of fibrosis and cancers [9]. Conversely, synthetic miRNAs administration can be used as a replacement therapy for EMT-/tumor-suppressor miRNAs that are commonly down-regulated in fibrosis and cancers [96]. Our present study may add a therapeutic option of targeting the caspases to control EMT and the pathophysiologic consequences.

## Materials and Methods

### *Chlamydia trachomatis* stock, animal infection and assessment of infertility

Stocks of *C*. *trachomatis* serovar L2, were propagated in HeLa cells and the purified elementary bodies (EBs) were titered as inclusion-forming units per milliliter (IFU/ml) by standard procedure [76]. Female TNF-alpha knockout (B6.129-TNFtm1LjoN7)(TNF-alpha^-/-^) wild-type C57BL/6) (TNF-alpha^+/+^) mice, 5–8 weeks old, were obtained from either Taconic Farms, Inc (Hudson, NY) or Jackson Laboratory (Bar Harbor, MA) Mice were infected intravaginally with 1x10^5^ IFU per mouse with *C*. *trachomatis* serovar L2 while under the long-acting anesthetic sodium pentobarbital (30 microgram/body weight)(Sigma-Aldrich, St Louis, MO) approximately 5 days after intramuscular administration of 2.5 microgram/mouse Depo Provera (medroxy-progesterone Acetate; Pfizer Inc, NY, NY). These conditions are a key factor to a successful mouse model of chlamydial genital infection using the human chlamydial strains [97]. Control mice were sham infected with PBS. The infection and infertility were assessed by tissue culture isolation of chlamydiae from cervico-vaginal swabs and enumeration of chlamydial inclusions by immunofluorescence method [76] and mating of mice with proven fertile males, as previously described [98]. In addition to fertility assessment, animals were visually and microscopically inspected and scored for uni- and bilateral hydrosalpinx or cysts, and presence of other abnormalities in the reproductive system [98,99]. All animal protocols were approved by the CDC Institutional Animal Care and Use Committee (IACUC).

### Immunohistochemistry

The murine oviduct epithelial cell line (C57epi.1) that supports the growth of chlamydiae was kindly provided by Dr Raymond Johnson, Indiana Univ., Indianapolis, IN, USA. Immunofluorescence staining of epithelial cells for EMT markers was performed on chlamydial-infected (MOI = 1) and non-infected monolayers by standard procedures after 72h of infection. Z-VAD-FMK and the control Z-FA-FMK were used in culture at 50 micromolar. Quantification of fluorescence was performed by scanning fluorescent-stained cells at 20X objective on a Nikon fluorescent microscope using the NIS-Elements Imaging Software version 3.20 (Nikon Instruments Inc, Melville, NY USA). The mean fluorescence intensity and standard deviations of 6 out of 10 scanned fields per slide were calculated automatically by the software. Immunohistochemistry (IHC) assays on sections of oviduct tissues were performed by using a polymer-based colorimetric indirect immunoalkaline phosphatase method with a polyclonal rabbit anti-T-cadherin antibody, as previously described [100].

### Micro-RNA Analysis by microarray and real time PCR

For miRNA microarray analysis, the Signosis’ miRNA Array IV service was used according to the company’s standard procedure (Signosis, Inc. Sunnyvale, CA), as previously described [3]. Briefly, total RNA was isolated from homogenized oviduct tissues and annealed to a biotin-UTP labeled oligonucleotide probe mixture corresponding to 540 randomly selected miRNAs which have been reported to play a role in inflammation, apoptosis and cancer by literature search [101]. After hybridization of biotin-UTP labeled probes, the miRNA expression arrays were detected by Streptavidin-HRP chemiluminescence. The chemiluminescent signals were acquired using the Alpha Innotech FluorChem FC2 imaging system. With the array assay, the expression of 540 miRNAs was profiled in the samples. Quantitative Real-Time PCR was used to validate the microarray data for selected miRNAs based on a minimum of two-fold increase or decrease among the experimental groups observed in the microarrays. There was a focus on miRNAs showing a decrease or increase in the oviducts from infected infertile mice, as compared to uninfected mice. For Real-time miRNA PCR, the miRNA-specific oligo mix was used instead of the random oligomix. The ligated products were eluted and mixed with miRNA specific qPCR primers and SYBR miRNA PCR buffer mix. The Real-Time PCR was conducted on the ABI 7700 system using TaqMan Small RNA assays (Applied Biosystems) and subjected to 35 PCR cycles: 95°C 15 seconds and 50 seconds. The small RNA U6 was used as an internal (endogenous) control and so the relative expression of miRNAs was normalized to U6 miRNA expression. Real-time PCR data were analyzed using the ddCT method by the standard procedure [101]. Information about miRNA target mRNA genes was obtained from several Web-accessible miRNA database searching programs, including http://www.microrna.org, http://www.miRBase.org, http://www.targetscan.org and published reports [102]. Experiments were repeated 4 times with at least 6 oviducts in each group.

### Proteomics Analysis

The quantitative differential proteomics with isobaric tags for relative quantitation (iTRAQ) labeling and LC-MS analysis was used to identify proteins that were differentially expressed in the oviducts from chlamydial infected mice, as previously described [103,104]. Briefly, proteins were extracted from oviducts and digested with trypsin, dried and reconstituted in 0.5 M TEAB then labeled with 4-plex iTRAQ according to the manufacturer’s AB SCIEX protocol (Applied Biosystens Inc., ABI, Foster City, CA), using the 114, 115, 116 and 117 iTRAQ tags. Labeled samples were pooled and subjected to a strong cation exchange and liquid chromatography, followed by mass spectrometry (MS) analysis of eluted fractions for protein identification and quantification. Protein/peptide identification and quantification were performed with the Protein Pilot Software version 4.5 and MASCOT, including the search of the database for murine or human proteins (http://www.uniprot.org). Only protein identified by at least 2 peptides and with a P-value < 0.05 were used for quantitation. The biological and functional properties of the proteins and their physiological and disease pathways were established through Elsevier software-Pathway Studio 10 (http://www.elsevier.com/online-tools/pathway-studio) and other specialized biomedical databases that include PubMed and Medline searches.

### Statistical Analysis

The data derived from different experiments were analyzed and compared by performing a one- or two-tailed *t* test, and the relationship between different experimental groupings was assessed by analysis of variance (ANOVA). Statistical significance was judged at *P< 0*.*05*.

### Ethics Statement

All animal protocols were approved by the CDC Institutional Animal Care and Use Committee (IACUC) under Protocol # 2605IGIMOUC-A1. The CDC IACUC is guided by Title 9, Chapter I, Subchapter A—Animal Welfare (USDA Regulations).
